# Endoscopic Endonasal Surgical Strategy for Skull Base Chordomas Based on Tumor Growth Directions: Surgical Outcomes of 167 Patients During 3 Years

**DOI:** 10.3389/fonc.2021.724972

**Published:** 2021-09-22

**Authors:** Jiwei Bai, Mingxuan Li, Yujia Xiong, Yutao Shen, Chunhui Liu, Peng Zhao, Lei Cao, Songbai Gui, Chuzhong Li, Yazhuo Zhang

**Affiliations:** ^1^Department of Neurosurgery, Beijing Tiantan Hospital, Capital Medical University, Beijing, China; ^2^Beijing Neurosurgical Institute, Capital Medical University, Beijing, China; ^3^Beijing Institute for Brain Disorders Brain Tumor Center, Beijing, China; ^4^China National Clinical Research Center for Neurological Diseases, Beijing, China; ^5^Key Laboratory of Central Nervous System Injury Research, Capital Medical University, Beijing, China

**Keywords:** endoscopic, endonasal, skull base, chordoma, treatment

## Abstract

**Background:**

Skull base chordomas (SBCs) are rare malignant bone tumors with dismal long-term local control. Endoscopic endonasal surgeries (EESs) are increasingly adopted to resect SBCs recently. Gross total resection (GTR) favors good outcomes. However, the SBCs often invade the skull base extensively and hide behind vital neurovascular structures; the tumors were challenging to remove entirely. To improve the GTR, we established a surgical strategy for EES according to the tumor growth directions.

**Methods:**

A total of 112 patients with SBCs from 2018 to 2019 were classified into the derivation group. We retrospectively analyzed their radiologic images and operation videos to find the accurate tumor locations. By doing so, we confirmed the tumor growth directions and established a surgical strategy. Fifty-five patients who were operated on in 2020 were regarded as the validation group, and we performed their operations following the surgical strategy to verify its value.

**Results:**

In the derivation group, 78.6% of SBCs invade the dorsum sellae and posterior clinoid process region. 62.5% and 69.6% of tumors extend to the left and right posterior spaces of cavernous ICA, respectively. 59.8% and 61.6% of tumors extend to the left and right posterior spaces of paraclival and lacerum ICA (pc-la ICA), respectively. 30.4% and 28.6% of tumors extended along the left and right petroclival fissures that extend toward the jugular foramen, respectively. 30.4% of tumors involved the foramen magnum and craniocervical junction region. The GTR was achieved in 60.8% of patients with primary SBCs in the derivation group. Based on the tumors’ growth pattern, pituitary transposition and posterior clinoidectomy techniques were adopted to resect tumors that hid behind cavernous ICA. Paraclival ICA transposition was used when the tumor invaded the posterior spaces of pc-la ICA. Lacerum fibrocartilage resection and eustachian tube transposition may be warranted to resect the tumors that extended to the jugular foramen. GTR was achieved in 75.0% of patients with primary SBCs in the validation group.

**Conclusion:**

Besides the midline clival region, the SBCs frequently grow into the eight spaces mentioned above. The surgical strategy based on the growth pattern contributes to increasing the GTR rate.

## Introduction

Skull base chordomas (SBCs) are traditionally considered to be histologically low-grade bony neoplasms. Although proliferation index ki-67 is low in most chordoma tissues except for poorly differentiated chordomas and dedifferentiated chordomas (1–3), SBCs show robust proliferation and invasive local growth capabilities. Furthermore, it was believed to be chemoresistant and resistant to traditional low-dose radiotherapy. The recurrence rate was high because of the unsatisfied resection rate and no effective adjuvant therapy available. Many advances have been achieved in medical treatment in recent years, and some drugs have shown effectiveness, but the overall response rate is still low in SBCs. Long-term survival and favorable neurological outcome continue to be challenging. The best available evidence supports a more aggressive surgical resection with negative microscopic margins; this radical resection correlates with improved local control and improved survival (4, 5). Although the resection rate seems improved with new equipment and endoscopic endonasal surgery (EES), the radical resection rate was still challenging for SBCs. Chordomas arising from the clivus are among the most challenging neoplasms for skull base surgeons (6). By summing up our recent surgical results and referring to previous literature, we established the surgical strategy that improved our resection rate lately.

## Materials and Methods

### Patients

This study includes all consecutive patients with SBCs between January 2018 and December 2020. They were treated by our single neuro-endoscopic group (which is directed by senior surgeons Y Zhang and S Gui). Pathologists confirmed the final diagnosis. All these patients underwent at least one EES in our ward. These patients were divided into two groups: the derivation group that includes the patients treated between January 2018 and December 2019 and the validation group that consists of the other patients treated in 2020. Our institutional review board approved this study. All patients signed informed consents before surgeries. We performed the final follow-up on May 2021 through WeChat or phone.

### Tumor Locations and Surgical Strategy

**Figure 1:**
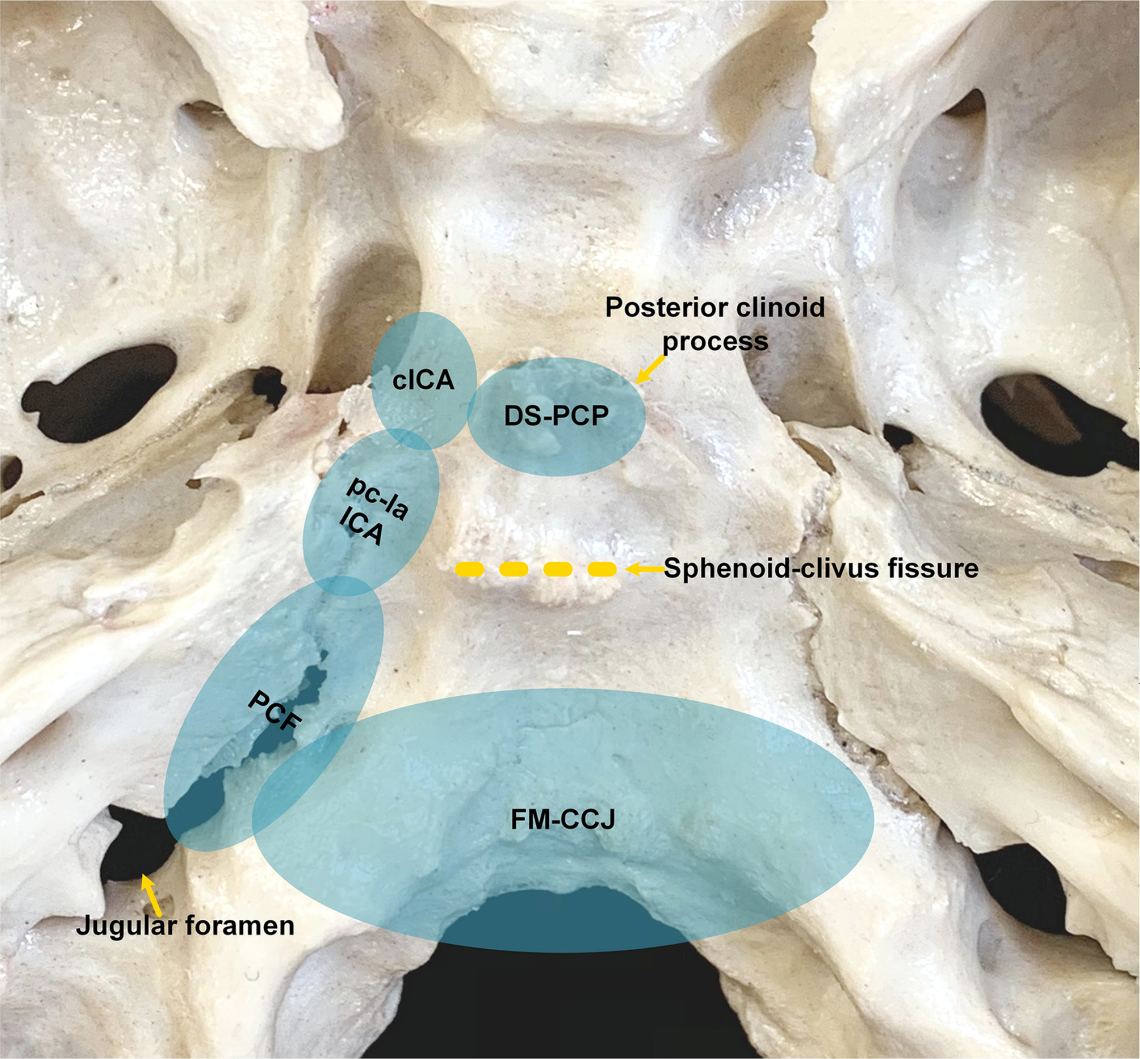
Anatomic spaces that skull base chordomas frequently invade. cICA, cavernous internal carotid artery; DS-PCP, dorsum sellae and posterior clinoid process; FM-CCJ, foramen magnum and craniocervical junction; PCF, petroclival fissure; pc-la ICA, paraclival and lacerum ICA.

J Bai, M Li, C Li, and Y Zhang retrospectively analyzed the pre- and postoperative images of patients in the derivation group. For patients in the validation group, all neurosurgeons examined the locations of the tumors during preoperative discussions, formulated surgical strategies, and judged the degree of resection during postoperative meetings. All patients were checked with CT on the same evening after surgery; unless the patient has serious complications or cannot safely perform MRI, the patients were usually scanned with MRI within 48 h. The degree of tumor resection is judged according to postoperative MR and CT images combined with surgical video, which includes gross-total resection (GTR), near-total resection (NTR), and partial resection (PR). GTR was defined as no residual soft tumor tissue during the operative inspection, and the surrounding bone was drilled to the normal-appearing bony structure, and no residual tumor was observed on postoperative images. No suspicious tumor was found during operation, and >90% tumoral resection on images was defined as NTR; <90% tumoral resection on images was defined as PR. Combined with literature reports on the location of chordoma (2, 6, 7), endoscopic anatomy (8), and our group’s experience in the EES treatment of chordoma (9), we found that chordoma has the characteristics of extending along the skull base sutures (**Figure 1**). Besides the central part of chordoma often located in the midline region of the clivus, the most commonly involved areas include the following eight spaces: dorsum sellae and posterior clinoid process (DS-PCP), bilateral posterior spaces of cavernous ICA (cICA), bilateral posterior spaces of paraclival and lacerum ICA (pc-la ICA), bilateral petroclival fissure spaces that extend toward the medial part of the jugular foramen, and foramen magnum and craniocervical junction (FM-CCJ). When retrospectively reviewing the images of the derivation group, we analyzed the distribution of chordomas in these eight spaces and confirmed the characteristics of chordoma extension along the sutures. A surgical strategy was formed; that is, the tumors in these eight areas were sequentially explored and removed during the operation. By analysis of surgical video and postoperative images, we evaluated our surgical strategy in the validation group.

Most SBCs can be resected through EES, and a few SBCs that extend beyond the inferior limit of EES need to be exposed by the combined endoscopic endonasal and transoral surgery. A vascularized nasal septal flap was prepared if possible, and then a standard sphenoidotomy was performed. We emphasize sufficient and safe tumor exposure, followed by exploring the eight aforementioned spaces according to the tumor growth directions. See below for the detailed descriptions. We mainly perform EES using the binostril four-hand technique, and three surgeons’ five-hand technique may be used when necessary. The surrounding bony structure, which was suspected to be eroded by the tumor, should be drilled to a healthy margin in a maximal safe manner.

#### Chordomas in Dorsum Sellae and Posterior Clinoid Process

**Figure 2:**
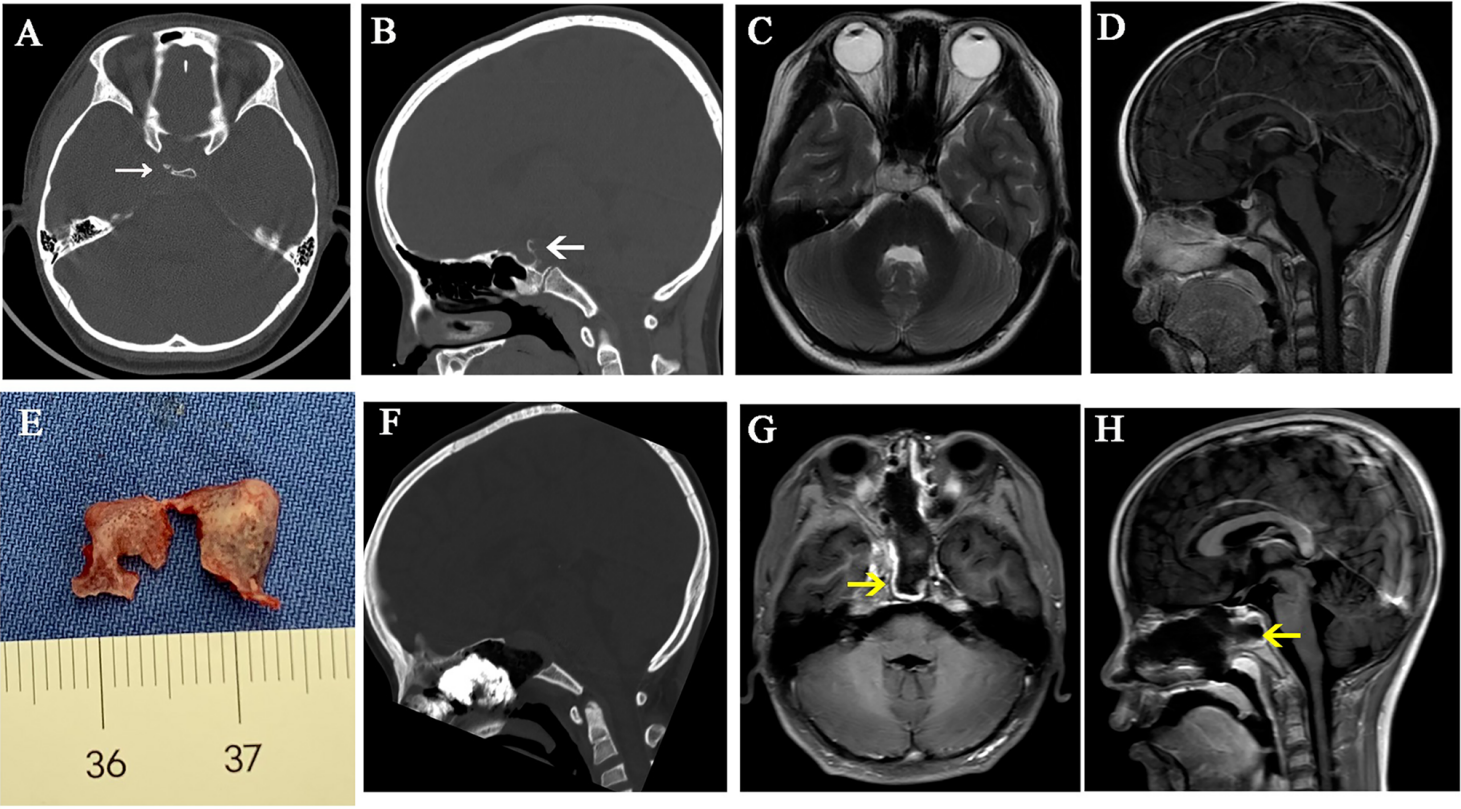
A case illustrates dorsum sellae and posterior clinoid process resection. **(A–D)** Preoperative CT and MR images show that the tumor is located in the upper and mid-clivus and erodes the dorsum sellae and posterior clinoid processes. **(E)** The dorsum sellae was split into two pieces and taken out with posterior clinoid processes. **(F–H)** Postoperative CT and MR images show that the dorsum sellae and posterior clinoid processes were removed. The white arrows represent the eroded dorsum sellae and posterior clinoid processes. The yellow arrows represent the vascularized nasal septal flap.

The bone of the sellar floor, middle clivus, and cavernous sinus were drilled. For small DS-PCP, the pituitary gland was elevated extradurally; then, dorsum sellae was split into two pieces in its middle part. After that, DS-PCPs were removed (**Figure 2**). When DS-PCP is large or adheres tightly with the dura mater, or the tumor invades into the intradural space and grows into the retro-infundibular region or inter-peduncular cistern, the interdural approach (10) is beneficial to resect DS-PCP and expose chordoma extensively.

#### Chordomas in Posterior Spaces of Cavernous ICA

**Figure 3:**
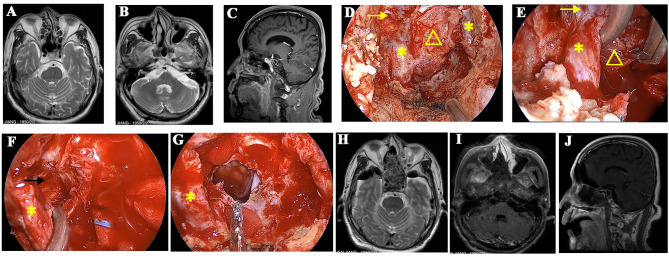
A recurrent chordoma locates in the upper and mid-clivus. **(A, B)** The axial MR images show that the tumor locates in the right posterior space of cavernous ICA and the right posterior space of paraclival ICA, respectively. **(C)** The sagittal view shows that the tumor locates in the upper and mid-clivus and invades into the posterior space of paraclival ICA. **(D)** Intraoperative image shows that the bone covering the paraclival and lacerum ICA was drilled. **(E)** The paraclival ICA is gently retracted, and the tumor behind ICA was resected with the angled instruments under the view of 45° endoscopy. **(F)** The posterior space of cavernous ICA is seen after tumor resection, and the margin is clear. **(G)** The tumor is totally removed, and brain stem is visible. **(H–J)** Postoperative MR images showed that the tumor was totally removed. The yellow arrows represent cavernous ICA. The yellow triangles represent the recurrent chordoma. The yellow asterisks represent paraclival ICA. The black arrow represents the posterior space of the cavernous ICA.

For certain chordoma, the extradural maneuver is sufficient to remove the small part hiding behind the cICA. When the chordoma extends even more laterally, and the extradural procedure is not adequate to visualize the tumor, we use the interdural pituitary transposition technique to access the posterior space of cICA (10). An angled endoscope (30° and 45°) and angled instruments are helpful for better visualization and maneuver. After tumor resection, the posterior wall of the cavernous sinus and superior-medial part of the petrous apex is visualized (**Figure 3**).

#### Chordomas in Posterior Spaces of Paraclival and Lacerum ICA

Suppose the tumor is soft and the lateral extension is not much. In that case, it can be resected with the angled endoscope and the angled instruments without drilling the bone covering the paraclival ICA. When the tumors extend more laterally, lateralization of the paraclival ICA is necessary to expose chordomas in posterior spaces of paraclival ICA. The medial and front bone covering paraclival ICA is drilled. Next, paraclival ICA transposition is made, and the bone posterior to ICA is drilled (**Figure 3**) (11). To mobilize the paraclival ICA more laterally, we can thoroughly drill the bone surrounding the lacerum ICA, including the lingual process.

#### Chordomas in Foramen Magnum and Craniocervical Junction Space and Petroclival Fissure

**Figure 4:**
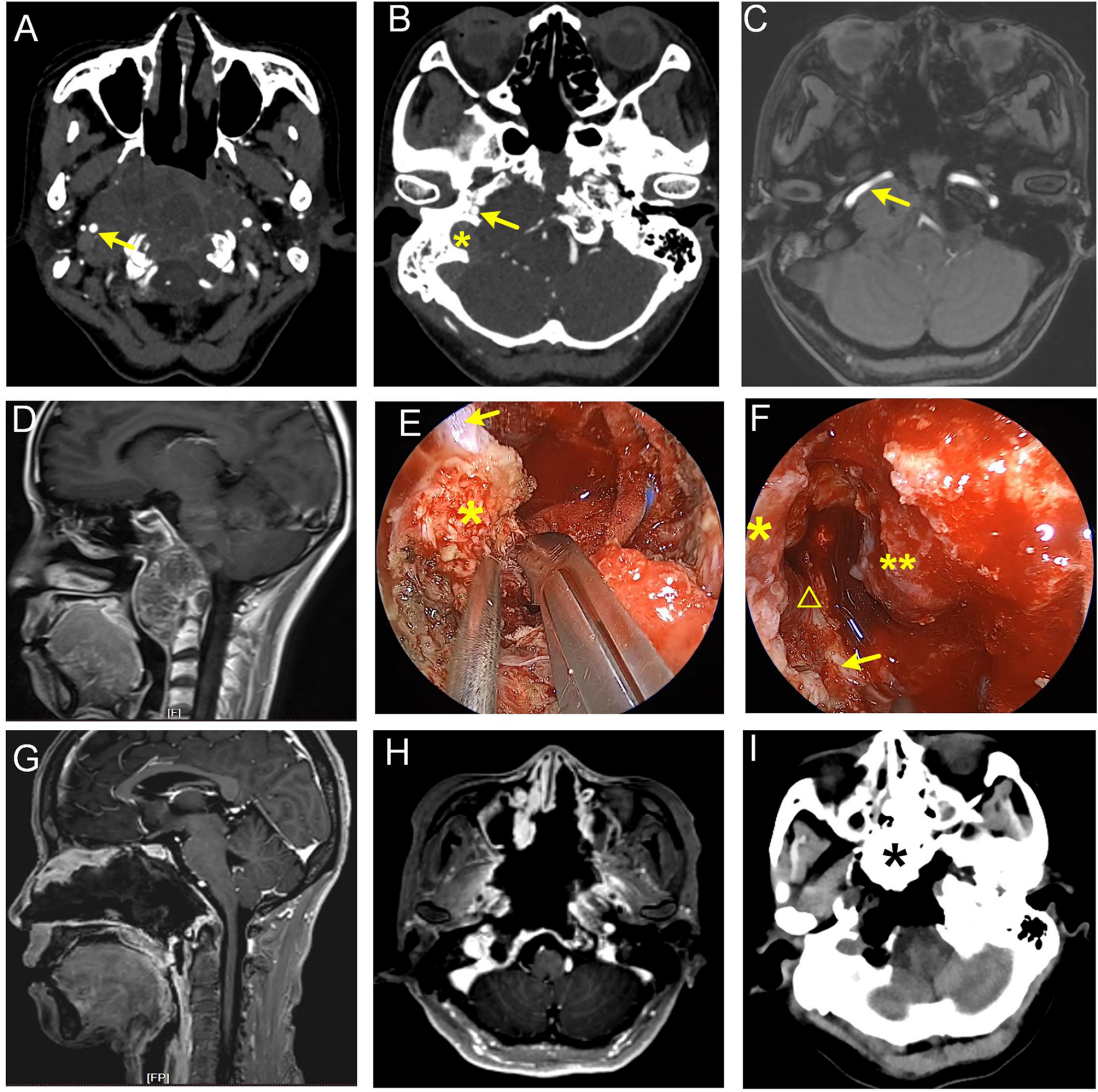
Giant recurrent chordoma involving petroclival fissure, jugular foramen, and craniocervical junction. **(A–D)** Preoperative images show that the tumor grew along the right petroclival fissure and destroyed petrous and clival bone. Parapharyngeal, petrous, lacerum, and paraclival ICA were involved. The arrow in **(A)** represents parapharyngeal ICA. Arrows in **(B, C)** represent petrous ICA, and the asterisk in **(B)** represents jugular foramen. **(E, F)** Intraoperative view. **(E)** Cut the hard scar and fibrocartilage following the course of the ICA with a sharp knife. The arrow denotes paraclival ICA. The asterisk indicates fibrocartilage tissue at lacerum. **(F)** Lacerum, petrous, and parapharyngeal ICA were exposed. Asterisk represents lacerum ICA, the triangle represents petrous ICA, the arrow represents parapharyngeal ICA, and the double-asterisk represents clival dura matter. **(G–I)** Postoperative images show that the tumor was near-totally removed. The asterisk in the panel I represents iodoform gauze.

The FM-CCJ space belongs to the lower clivus. We use a similar strategy to deal with tumors in petroclival fissure spaces, which often extend toward the medial part of the jugular foramen. When the chordoma locates in the midline area of the lower clivus, and the tumor volume is small, the tumor can be accessed by the inverted U-shaped rhinopharyngeal mucosal flap. The rhinopharyngeal mucosa and the basipharyngeal fascia, as well as longus capitis muscle and rectus capitis muscle, are inferiorly mobilized. Thus, the entire inferior clivus can be accessed, including the pharyngeal tubercle, anterior border of the foramen magnum, the anterior ring of C1, and the craniocervical junction (12). If the hard palate is high and hampers the caudal surgical corridor, the rear of the hard palate may be drilled to increase the nasopalatine angle and the caudal exposure. Generally, EES can reach the cervical spine of the C2 level. When the chordomas extend more inferiorly, a combined transoral approach or pure transoral approach may be performed. When the chordomas grow along the petroclival fissure significantly and extend toward jugular foramen, inferior turbinate, pterygoid process, eustachian tube, and parapharyngeal muscles may limit the tumor exposure laterally. In this circumstance, a prelacrimal recess approach is performed firstly (13), then lateral nasal wall flap is elevated and displaced into maxillary sinus; sequentially, pterygoid base and pterygoid plates are drilled, and the fibrocartilage around lacerum ICA and the eustachian tube are cut. By doing so, petrous ICA can be well exposed, and a more lateral view to the jugular foramen is achieved (**Figure 4**) (11).

#### Skull Base Reconstruction

**Figure 5:**
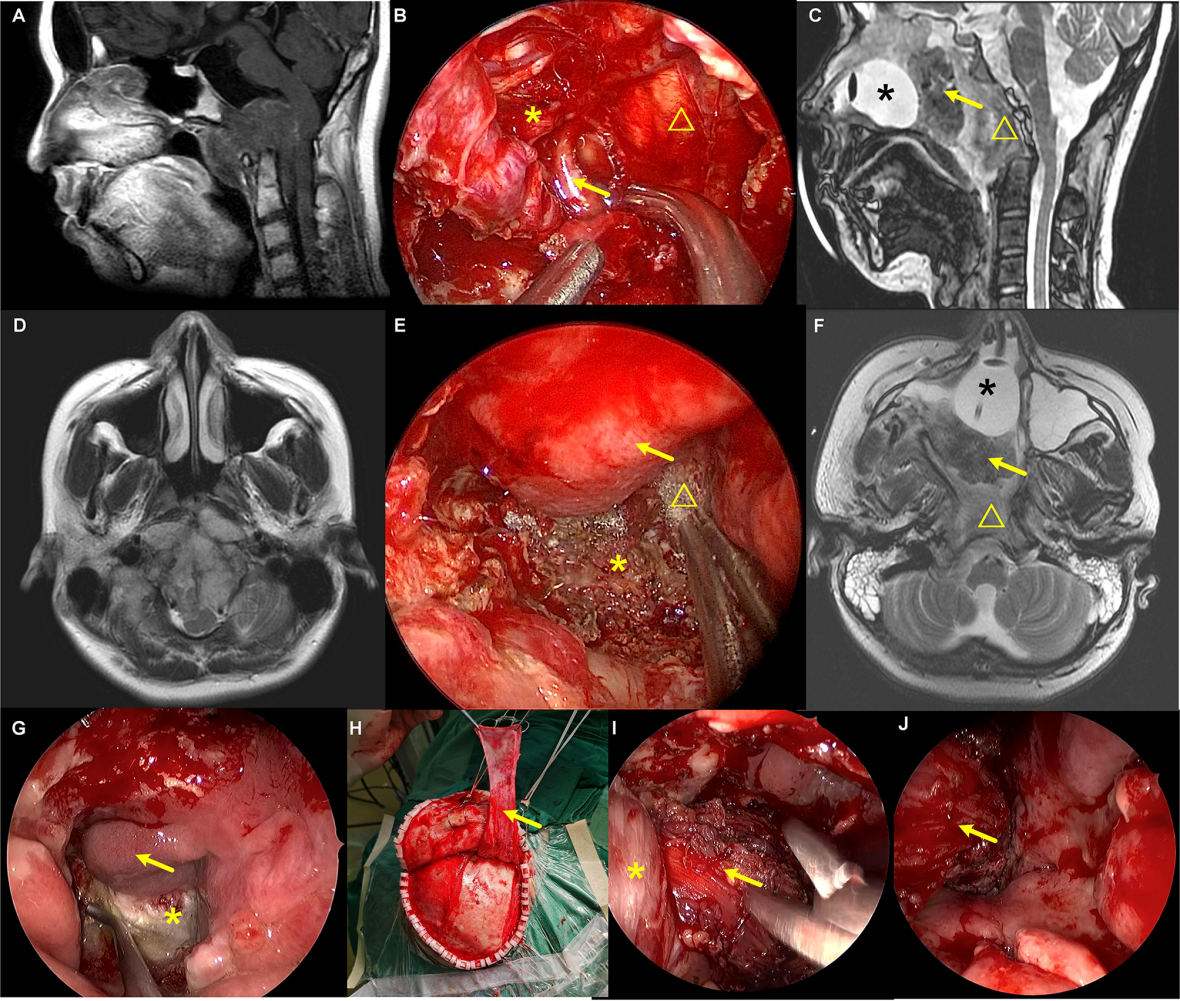
The repair of a refractory CSF leakage in the lower clivus and craniocervical junction with temporalis muscle–fascia–periosteum flap. **(A, D)** Preoperative images showed that the tumor was located in the lower clivus and extended into the cervical spinal canal. The medulla oblongata was pushed by the tumor and severely deformed. **(B, E)** Intraoperative view of the tumor resection. **(B)** Arrow represents the right vertebral artery. Asterisk represents the vagus nerve. Triangle represents the medulla oblongata. **(E)** Skull base reconstruction with artificial dura mater, autologous fat tissue, fascia lata, and pedicled nasoseptal flap. Arrow represents the nasoseptal flap. Asterisk represents the tip of the odontoid. Triangle represents the fascia lata. **(C, F)** Postoperative images after the tumor resection and three CSF leakage repairing surgeries. Asterisk represents the balloon of the urethral catheter. Arrow represents the iodoform gauze. Triangle represents the temporalis muscle flap. **(G–J)** Repairing CSF leakage with pedicled vascularized temporalis muscle–fascia–periosteum flap after two failed repairing surgeries. **(G)** Arrow represents the pedicled nasoseptal flap. Asterisk represents the fascia lata used for skull base reconstruction in the tumor resection surgery, which was found to be necrosis. **(H–J)** Arrow represents the pedicled temporalis muscle–fascia–periosteum flap. Asterisk represents the lateral pterygoid muscle.

A vascularized nasoseptal flap is extremely useful for skull base reconstruction in patients with primary chordoma. The nasoseptal flap is big enough to reconstruct the defect in the upper and middle clivus. However, it is too small to cover the more extensive defects in the lower clivus and the craniocervical junction. In this circumstance, an extended vascularized flap was harvested. Nasal floor mucoperiosteum was included in the vascularized flap. Intradural collagen was used as an inlay graft; then, autologous fat tissue was used as the second layer to eliminate dead space and avoid the ventral herniation of the brainstem through the dural defect (14). Then, autologous fascia lata covered onlay, followed by the extended vascularized nasoseptal flap and the inverted-U flap. For recurrent chordomas after EES, harvesting vascularized nasoseptal flap is difficult. Therefore, we will suture fat pad to dura mater using 6-0 prolene sutures with interrupted suture technique, which is an essential step for reconstruction. For the patient with radiotherapy history and refractory CSF leakage, the CSF leakage was successfully resolved with a vascularized temporalis muscle–fascia–periosteum flap. The temporalis muscle–fascia–periosteum flap was harvested through an open frontotemporal incision with its pedicle locating on the coronal process of the mandible. The flap includes the temporalis muscle, the temporalis fascia, and the periosteum between the supratemporal line and the skin incision. Then, the temporalis muscle–fascia–periosteum flap was displaced through the maxillary sinus to cover the defect (**Figure 5**).

### Statistics

SPSS software (version 19.0, IBM Inc.) was used for the statistical analysis. Student’s *t*-test was used for continuous variables (shown as mean ± SD); chi-square test or Mann–Whitney *U* test was applied for categorical variables (presented as number, %). All tests were two-sided, and *p* < 0.05 was regarded as statistically significant.

## Results

### Patient Characteristics

**Figure 6:**
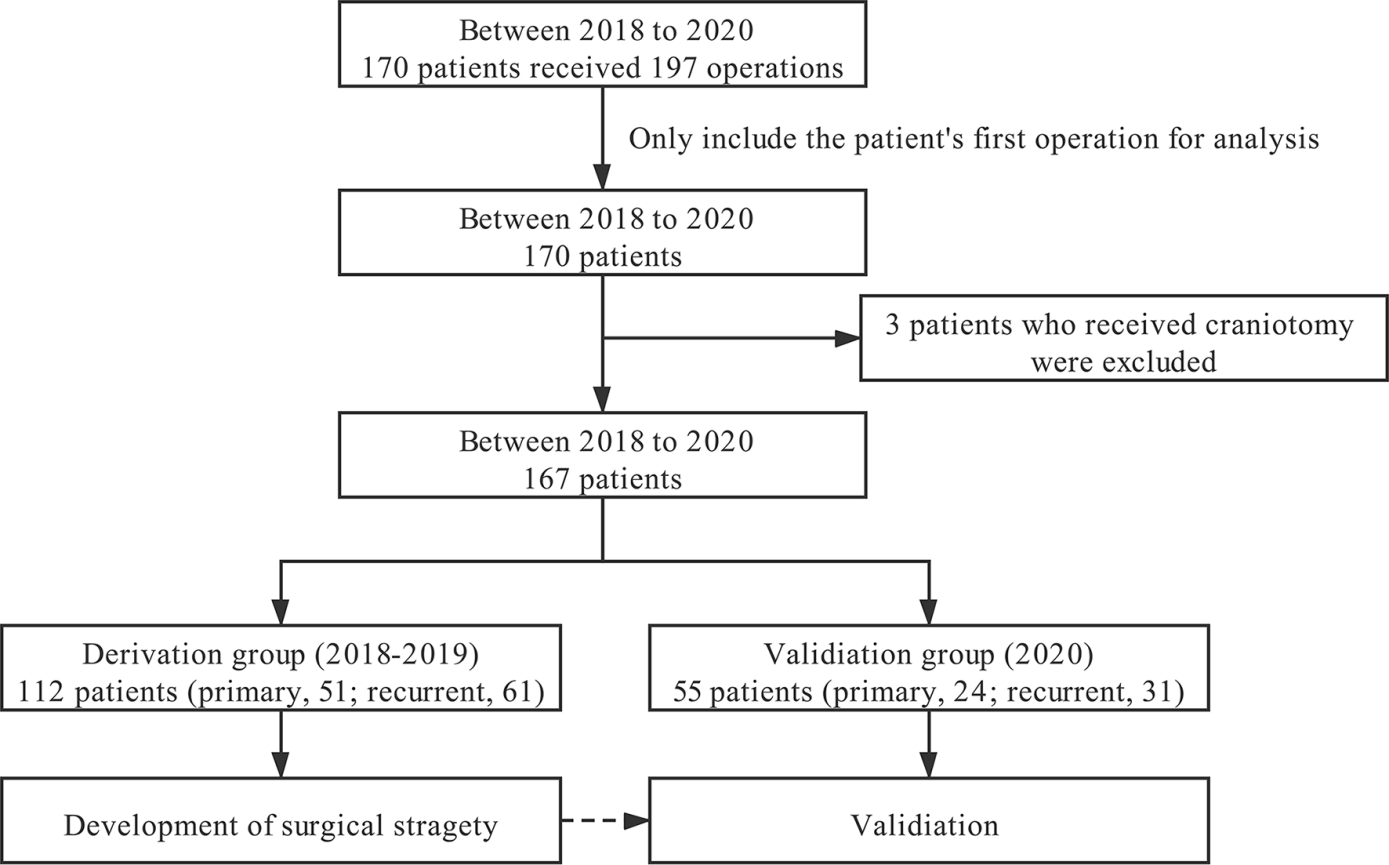
Flowchart for patient selection.

During the study period, 170 patients received 197 operations, namely, 100 males and 70 females. Two patients underwent staged surgery; among them, one patient had EEA first, followed by far lateral approach craniotomy, and the other had two EEAs. This study only included their first operation information in our institute for further analysis. Three patients who received craniotomy were excluded from the current study. Since some chordomas in the craniocervical junction and upper cervical segment require a combination of transoral approach (three patients), which have similar treatment strategies to the transnasal approach, these patients were also included in this study. Therefore, 167 patients with chordoma were eventually included in the study, with 112 patients in the derivation and 55 patients in the validation group (**Figure 6**).

The age of the entire cohort was 43.12 ± 17.53 years (mean ± SD), and there was no difference in age distribution between the derivation group and the validation group (43.03 ± 17.20 *versus* 43.31 ± 18.34 years, *p* = 0.922). Ninety-nine (59.3%) males and 68 (40.7%) males were included, and no difference in gender was observed between the two groups (male/female, 68/44 *versus* 31/24, *p* = 0.591). The derivation group included 51 (45.5%) primary patients and 61 (54.5%) recurrent patients, and the validation group included 24 (43.6%) primary patients and 31 (56.4%) recurrent patients (*p* = 0.817) (**Table 1**).

**Table 1:** Demographic and clinical characteristics of skull base chordoma patients.

Variables	Number of patients (%)			*p*-value
	Total	Derivation group	Validation group	
Age (mean ± SD)	43.12 ± 17.53	43.03 ± 17.20	43.31 ± 18.34	0.922
Sex				0.591
Male	99 (59.3%)	68 (60.7%)	31 (56.4%)	
Female	68 (40.7%)	44 (39.3%)	24 (43.6%)	
Primary or recurrent tumor				0.817
Primary	75 (44.9%)	51 (45.5%)	24 (43.6%)	
Recurrent	92 (55.1%)	61 (54.5%)	31 (56.4%)	
Symptoms				
Diplopia	76 (45.5%)	57 (50.9%)	19 (34.5%)	
Headache/neck pain	45 (26.9%)	32 (28.6%)	13 (23.6%)	
Visual impairment	38 (22.8%)	29 (25.9%)	9 (16.4%)	
Blepharoptosis	16 (9.6%)	12 (10.7%)	4 (7.3%)	
Dysphagia	15 (9.0%)	9 (8.0%)	6 (10.9%)	
Nasal obstruction	13 (7.8%)	9 (8.0%)	4 (7.3%)	
Hearing loss	9 (5.4%)	5 (4.5%)	4 (7.3%)	
Limb weakness	8 (4.8%)	5 (4.5%)	3 (5.5%)	
Dizziness	7 (4.2%)	4 (3.6%)	3 (5.5%)	
Facial numbness	5 (3.0%)	2 (1.8%)	3 (5.5%)	
Accident	16 (9.6%)	9 (8.0%)	7 (12.7%)	
Preoperative radiotherapy	37 (22.2%)	23 (20.5%)	14 (25.5%)	0.472
Postoperative radiotherapy	49 (29.3%)	26 (23.2%)	23 (41.8%)	**0.013**
Radiosurgery	11	8	3	
IMRT	10	6	4	
Proton beam therapy/Carbon ion therapy	25	9	16	
Unclear	3	3	0	

IMRT, Intensity-modulated radiation therapy.

Bold values indicate p < 0.05.

Diplopia caused by VI cranial nerve palsy [76 (45.5%) patients], which exhibits double vision increasing when looking lateral, was the most common clinical symptom, followed by headache/neck pain [45 (26.9%) patients], visual impairment [38 (22.8%) patients], blepharoptosis [16 (9.6%) patients], and dysphagia [15 (9.0%) patients]. The symptom distribution of the derivation group and the validation group was similar (**Table 1**).

There were 37 (22.2%) patients with a history of preoperative radiotherapy, and no significant difference was identified between the two groups [23 (20.5%) patients *versus* 14 (25.5%) patients, *p* = 0.472]. As the availability of different types of radiotherapy in different institutions and the high cost of particle radiation therapy, the patients received no uniform postoperative radiotherapy. Forty-nine (29.3%) patients received postoperative radiotherapy, including 11 cases with radiosurgery, 10 cases with intensity-modulated radiation therapy, and 25 cases with particle radiation therapy (proton beam therapy/carbon ion therapy). The rate of receiving postoperative radiotherapy was higher in the validation group (41.8%) compared to the derivation group (23.2%) (*p* = 0.013). In addition, proton beam therapy/carbon ion therapy accounted for the majority (16/23 patients) of the validation group (**Table 1**).

### Tumor Locations and Involved Anatomical Spaces

According to the classification of upper, middle, and lower clival, the most common locations are the upper-middle clival region [77 (46.1%) patients; derivation group, 54 (48.2%) patients; validation group, 23 (41.8%) patients] and the whole clival region [52 (31.1%) patients; derivation group, 36 (32.1%) patients; validation group, 16 (29.1%) patients]. Twenty-five (15.0%) patients showed a tumor location of both cranio-vertebral joint and clival [derivation group, 15 (13.4%) patients; validation group, 10 (18.2%) patients]. There was no significant difference in the different segments of clivus between the two groups (*p* = 0.409) (**Table 2**).

**Table 2:** The detailed tumor locations and involved anatomical spaces.

Variables	Number of patients (%)			*p*-value
	Total	Derivation group	Validation group	
Tumor locations				0.409
Upper clivus	0 (0.0%)	0 (0.0%)	0 (0.0%)	
Upper-middle clivus	77 (46.1%)	54 (48.2%)	23 (41.8%)	
Middle clivus	2 (1.2%)	0 (0.0%)	2 (3.6%)	
Middle-lower clivus	5 (3.0%)	3 (2.7%)	2 (3.6%)	
Whole clivus	52 (31.1%)	36 (32.1%)	16 (29.1%)	
Cranio-vertebral joint	5 (3.0%)	3 (2.7%)	2 (3.6%)	
Cranio-vertebral joint and clivus	25 (15.0%)	15 (13.4%)	10 (18.2%)	
Other location	1 (0.6%)	1 (0.9%)	0 (0.0%)	
Involved anatomical spaces				
DS-PCP	126 (75.4%)	88 (78.6%)	38 (69.1%)	0.181
Left cICA	102 (61.1%)	70 (62.5%)	32 (58.2%)	0.591
Right cICA	117 (70.1%)	78 (69.6%)	39 (70.9%)	0.867
Left pc-la ICA	98 (58.7%)	67 (59.8%)	31 (56.4%)	0.670
Right pc-la ICA	100 (59.9%)	69 (61.6%)	31 (56.4%)	0.516
Left PCF	50 (29.9%)	34 (30.4%)	16 (29.1%)	0.867
Right PCF	51 (30.5%)	32 (28.6%)	19 (34.5%)	0.431
FM-CCJ	55 (32.9%)	34 (30.4%)	21 (38.2%)	0.312

DS-PCP, dorsum sellae and posterior clinoid process; cICA, cavernous internal carotid artery; pc-la ICA, paraclival and lacerum internal carotid artery; PCF, petroclival fissure; FM-CCJ, foramen magnum and craniocervical junction.

DS-PCP [126 (75.4%) patients; derivation group, 88 (78.6%) patients; validation group, 38 (69.1%) patients; *p* = 0.181] was the most common involved anatomical area, followed by right cICA [117 (70.1%) patients; derivation group, 78 (69.6%) patients; validation group, 39 (70.9%) patients; *p* = 0.591] and left cICA [102 (61.1%) patients; derivation group, 70 (62.5%) patients; validation group, 32 (58.2%) patients; *p* = 0.867]. Left pc-la ICA and right pc-la ICA involvement were observed in 98 (58.7%) and 100 (59.9%) patients, respectively, and no distribution difference was identified between two groups (59.8% *versus* 56.4%, *p* = 0.670 and 61.6% *versus* 56.4%, *p* = 0.516). As for the petroclival fissure region, 50 (29.9%) patients had left petroclival fissure involvement [derivation group, 34 (30.4%) patients; validation group, 16 (29.1%) patients; *p* = 0.867], 51 (30.5%) patients had right petroclival fissure involvement [derivation group, 32 (28.6%) patients; validation group, 19 (34.5%) patients; *p* = 0.431], and 55 (32.9%) patients with tumors involved FM-CCJ, of which 34 (30.4%) patients were in the derivation group and 21 (38.2%) patients were in the validation group (*p* = 0.312) (**Table 2**). In summary, the involved anatomical area showed no difference between the two groups (all *p* > 0.05).

### Extent of Resection

The most intriguing result of the current study was the extent of resection. As shown in **Table 3**, patients with GTR, NTR, and PR were 73 (43.7%), 66 (39.5%), and 28 (16.8%), respectively. Interestingly, we observed a higher degree of resection in the validation group than in the derivation group (*p* = 0.003). Specifically, the GTR rate was 58.2% in the validation group, while it was 36.6% in the derivation group.

**Table 3:** Extent of resection in the derivation group and the validation group.

Extent of resection	Number of patients (%)			*p*-value
	Total	Derivation group	Validation group	
Whole cohort				**0.003**
GTR	73 (43.7%)	41 (36.6%)	32 (58.2%)	
NTR	66 (39.5%)	47 (42.0%)	19 (34.5%)	
PR	28 (16.8%)	24 (21.4%)	4 (7.3%)	
Primary cohort				0.227
GTR	49 (65.3%)	31 (60.8%)	18 (75.0%)	
NTR	21 (28.0%)	16 (31.4%)	5 (20.8%)	
PR	5 (6.7%)	4 (7.8%)	1 (4.2%)	
Recurrent cohort				**0.001**
GTR	24 (26.1%)	10 (16.4%)	14 (45.2%)	
NTR	45 (48.9%)	31 (50.8%)	14 (45.2%)	
PR	23 (25.0%)	20 (32.8%)	3 (9.7%)	

GTR, Gross total resection; NTR, Near-total resection; PR, Partial resection.

Bold values indicate p < 0.05.

Next, we stratified patients into the primary and recurrent cohorts for analysis. In the primary cohort, 18 (75.0%) patients received GTR in the validation group, which is higher than that in the derivation group (31, 60.8% patients), though the difference was not significant (*p* = 0.227). In the recurrent cohort, a significant difference was observed between the two groups (*p* = 0.001). The validation group showed a higher GTR rate and a lower PR rate than the derivation group (GTR rate, 45.2% *versus* 16.4%; PR rate, 9.7% *versus* 32.8%) (**Table 3**).

We also analyzed the extent of resection in patients with different tumor locations. Interestingly, we found a trend of higher resection rates in the validation group compared to those in the derivation group regardless of the tumor locations (**Table 4**). Of note, for the whole clival chordomas, a significant difference was observed; the GTR, NTR, and PR rates were 57.4%, 35.2%, and 7.4% in the derivation group, and 65.2%, 34.8%, and 0.0% in the validation group, respectively (*p* = 0.001). In addition, for chordomas in the craniovertebral joint and clivus, a higher resection rate was identified in the validation group than the derivation group (GTR rate, 30% *versus* 6.7%). However, the *p*-value (0.140) was not significant. It may arise due to the limited patients in each group (15 patients and 10 patients).

**Table 4:** Extent of resection of skull base chordoma in different locations between the two groups.

Extent of resection	Whole cohort (%)			Derivation group (%)			Validation group (%)			*p*-value
	GTR	NTR	PR	GTR	NTR	PR	GTR	NTR	PR	
Tumor locations									
Upper clivus	0	0	0	0	0	0	0	0	0	NA
Upper-middle clivus	46 (59.7%)	27 (35.1%)	4 (5.2%)	31 (57.4%)	19 (35.2%)	4 (7.4%)	15 (65.2%)	8 (34.8%)	0 (0.0%)	0.405
Middle clivus	2 (100.0%)	0 (0.0%)	0 (0.0%)	0	0	0	2 (100.0%)	0 (0.0%)	0 (0.0%)	NA
Middle-lower clivus	2 (40.0%)	3 (60.0%)	5 (0.0%)	1 (33.3%)	2 (66.7%)	0(0.0%)	1 (50.0%)	1 (50.0%)	0 (0.0%)	NA
Whole clivus	17 (32.7%)	26 (50.0%)	9 (17.3%)	7 (19.4%)	20 (55.6%)	9 (25.0%)	10 (62.5%)	6 (37.5%)	0 (0.0%)	**0.001**
Craniovertebral joint	2 (40.0%)	3 (60.0%)	0 (0.0%)	1 (33.3%)	2 (66.7%)	0(0.0%)	1 (50.0%)	1 (50.0%)	0 (0.0%)	NA
Craniovertebral joint and clivus	4 (16.0%)	7 (28.0%)	14 (56.0%)	1 (6.7%)	4 (26.7%)	10 (66.7%)	3 (30.0%)	3 (30.0%)	4 (40.0%)	0.140
Other location	0 (0.0%)	0 (0.0%)	1 (100.0%)	0 (0.0%)	0 (0.0%)	1(100.0%)	0	0	0	NA

GTR, Gross total resection; NTR, Near-total resection; PR, Partial resection; NA, not applicable.

Bold values indicate p < 0.05.

### Complications

Twenty-two (13.2%) patients suffered from surgical complications in the current study (derivation group, 15 patients; validation group, 7 patients). The most common complications were cerebrospinal fluid leakage (9, 5.4% patients; among them, 4 patients with primary SBC and 5 patients with recurrent tumor), intracranial infection (5, 3.0% patients), and cranial nerve defect (5, 3.0% patients). In addition, respiratory dysfunction was observed in four (2.4%) patients, and two (1.2%) patients suffered from carotid artery injury. Of note, no significant difference in complication rate was observed between the discovery and validation groups (13.4% *versus* 12.7%, *p* = 0.905) (**Table 5**).

**Table 5:** Surgical complications in skull base chordoma patients.

Variables	Number of patients			*p*-value
	Total	Derivation group	Validation group	
Complications (%)	22 (13.2%)	15 (13.4%)	7 (12.7%)	0.905
Cerebrospinal fluid leakage	9 (5.4%)	5 (4.5%)	4 (7.3%)	
Intracranial infection	5 (3.0%)	4 (3.6%)	1 (1.8%)	
Cranial nerve defect	5 (3.0%)	3 (2.7%)	2 (3.6%)	
Respiratory dysfunction	4 (2.4%)	4 (3.6%)	0 (0.0%)	
Carotid artery injury	2 (1.2%)	2 (1.8%)	0 (0.0%)	
Hypopituitarism	2 (1.2%)	1 (0.9%)	1 (1.8%)	
Paralysis	1 (0.6%)	0 (0.0%)	1 (1.8%)	

### Follow-Up

One patient died due to surgical complications, and three patients were lost to follow-up. The dead patient has undergone three operations previously and RT and chemotherapy history. He underwent the fourth operation for debulking and died of brain extensive cerebral ischemia due to continuing low blood pressure during operation, related to the patient’s poor base performance status. The remaining 163 patients (derivation group, 109 patients; validation group, 54 patients) were regularly followed up with a mean time of 18.7 months (derivation group, 23.7 months; validation group, 8.4 months) (range, 2–39 months). Seventy-five (46.0%) patients [derivation group, 65 (59.6%) patients; validation group, 10 (18.5%) patients] suffered from tumor progression or recurrence and 25 (15.3%) patients [derivation group, 24 (22.0%) patients; validation group, 1 (1.9%) patients] died during the follow-up.

## Discussion

The incidence of SBCs is extremely low, and its mortality and recurrence rate remain high. Surgical treatment is the initial and indispensable treatment for SBCs. The resection quality is associated with the patients’ long-term outcomes (15). However, to our knowledge, only limited studies with a relatively large patient number reported the surgical experience and outcomes (3, 7, 16–19). Given their ideal midline location and ventral to the skull base dura mater (2), SBCs are increasingly treated through EES with the development of neuroendoscopic instruments, surgical experience acquisition, and surgical technique advancement (20–23). During the present study, three patients underwent open surgeries in 2018 and 2019, and one patient experienced a staged craniotomy after EES in 2020. The EES contributes to a high tumor removal and symptom control rate, with low morbidity and high quality of life (2). In our opinion, further improvement of the surgical resection extent and safety is the focus of EES. However, the learning curve is remarkable in EES (2, 19). Previous studies (2, 7, 9) included the patients during a relatively long period; therefore, the learning curve significantly impacted tumor resection (20). Our group started EES in 1998 and has extensive SBC surgical experience (9). Only the SBC patients treated from 2018 to 2020 were included in the present study; hence, the impact of the learning curve was minimized. Furthermore, the adjuvant equipment (such as intraoperative Doppler, neuronavigation, and neuromonitoring) and materials for skull base reconstruction (such as collagen graft and iodoform gauze) did not change in the last 3 years and had no impact on the surgery. Therefore, the present study is suitable for exploring the relationship between surgical strategy and resection rate.

Several classifications of SBCs have been proposed to guide the operation or judge the prognosis. Based on open surgery experience and SBCs’ extension patterns, Amelfty et al. proposed a surgical classification to clarify the best microsurgical approach (5). At the same time, EES was beginning to be used to resect SBC (24). However, EES has rapidly increased in SBC surgeries in recent years, and EES can access cervical 2 as the most inferior limit of dissection; the microsurgical classification cannot direct EES anymore. In 2014, Fernandez-Miranda et al. described the EES treating SBCs according to dividing clivus into three parts (i.e., upper clivus, mid-clivus, and lower clivus) (6).

Similarly, our group proposed a clinical classification for EES in SBCs in 2016 (9). The results suggested that EES may improve the resection degree and surgical efficacy of SBCs. The limitation of our previous classification is that it ignores the tumor-extending direction. In 2018, Sekhar et al. proposed a preoperative grading system that is helpful in predicting resection and outcome (18). The tumor site has the highest weight in the score. The more sites were involved, the score was higher, and the progression-free survival was shorter. Sekhar’s grading system indicated that the growth pattern of chordoma plays a vital role in guiding surgery and judging prognosis. Most recently, Wang et al. proposed that the SBCs were classified into four types based on the analysis of 55 patients (25). This classification introduced a new line that connects the anterior part of the sellar floor with the intersection of the sphenoid floor plane and the dorsal margin of the clivus. Although Wang’s classification was based on the tumor origin and growth pattern, the category does not use the anatomical sites that surgeons are familiar with, so it is impossible to direct surgical strategy accurately, which is its main disadvantage. Furthermore, it is difficult to classify tumors in patients with multiple recurrent tumors because their skull base structures are complex, and the affected scope is too broad (25). In the present study, we found that the tumors extend along the bone sutures after analyzing the images and surgeries of SBC patients in the derivation group. This feature was also hypothesized in the study of another craniotomy team (7). We further confirmed that the hypothesis is reasonable by analyzing the images and surgeries of SBC patients in the validation group. In addition, we designed and validated our surgical strategies based on the spaces of the SBCs’ eight most common extending areas. These eight spaces have little anatomic variations, even in patients with multiple recurrent chordomas or extensive invasion chordomas.

Traditionally, EES is best used for midline lesions that lack significant lateral extension. We found that almost all SBCs are involved in at least one of the eight spaces mentioned above besides the main body of the tumors located in the midline region of the skull base. These eight spaces are difficult to access directly and are the most common spaces for residual tumors (25). Previously, we performed surgeries following the “tumor-based” resection strategy. Small residual tumors were sometimes found in the spaces mentioned above on the postoperative MRI, although all the tumors seemed to be removed during the operation. Benefiting from recognizing the potential extending directions of SBCs, we perform “tumor-based” and “anatomy-based” resection now, i.e., to explore the possible hidden tumors in the eight spaces after resection of the main tumor. Although we found no statistical difference between the derivation and validation groups in the GTR of primary SBCs, it showed an increasing trend. It may be limited by the small sample size of primary SBCs. Furthermore, it may correlate with the relatively apparent anatomy in primary SBCs, and the endoscopic surgical technique for primary SBCs resection in our group is mature. The operations of recurrent SBCs were challenging. The recurrent SBC’s anatomy is complex, and the texture is more rigid. The tumor may be divided into multi-cavities by smooth fiber tissue membrane, resulting in the surgeon mistakenly regarding the membrane as the tumor margin. However, it is exciting that our new surgical strategy increased the GTR significantly in recurrent SBCs. In summary, our strategy is suitable for both primary SBCs and recurrent SBCs.

This strategy emphasizes using the corresponding EES technique according to the different spaces. The standard transnasal sphenoid approach was performed to expose the sphenoid sinus and middle clivus and explore the tumor according to the eight possible extending areas. SBCs are osteo-destructive tumors that erode the surrounding bony structures; therefore, bone margin removal is critical for radical resection (5, 7). It is necessary to visualize the bone margin directly with a 0° endoscope to drill the lateral bony tumors. However, the soft tumors may be removed with angled instruments in the view field of angled endoscopy. Every blind spot should be carefully reviewed after tumor resection to confirm no residue tumors.

EES provides adequate exposure to the clivus’ upper, middle, and lower parts (22). The upper clivus is formed by the dorsum sellae and the posterior clinoid processes (6, 9). Wang’s study showed that residual tumors were mainly in the cavernous sinus or the rear upper part of the dorsum sellae (25). In our experience, SBCs often erode the dorsum sellae and the posterior clinoid processes, and the resection rate can be improved by posterior clinoidectomy. When the dorsum sellae is small, the dorsum sellae and posterior clinoid processes can be removed extradurally by splitting the dorsum sellae into two pieces and separating them from the dura mater. The extradural approach is relatively safe because this technique does not maneuver the cavernous sinus; the pituitary gland can be superior elevated without a significant bleeding risk (26). When the dorsum sellae is large, or the adhesion between the dura mater and posterior clinoid processes is tight, the intradural pituitary transposition is an alternative for posterior clinoidectomy (21). However, this technique has the potential risk of damaging the pituitary gland. Therefore, we favor the interdural transcavernous approach to perform posterior clinoidectomies (10). Even for the SBCs that invade the retro-infundibular region and interpeduncular cistern, EES can safely remove it following the corridor after the dorsum sellar resection and posterior clinoidectomy.

The middle clivus extends vertically from the sellar floor to the floor of the sphenoid body, which is at the level of paraclival ICA and lacerum ICA (6, 9). In most cases, manipulation with angled instruments combined with angled endoscopy allows for the resection of the soft SBCs located behind the paraclival ICA. If the tumors extend more laterally, the pterygoid tubercle and lingual process should be drilled extensively, and then the paraclival can be moved laterally (27). With the eustachian tube resection, more lateralization of ICA can be achieved (28). The most medial aspect of the petrous apex also locates behind the ICA (6). SBCs often extend laterally and inferiorly toward the petrous apex and foramen lacerum, and a sublacerum approach is helpful for complete resection of the petrous apex tumor component (27). To expose more laterally, EES combined with the transmaxillary operation (Denker procedure or Caldwell-Luc procedure) is helpful to access the petrous apex (12). Recently, Patel et al. suggested that a contralateral transmaxillary corridor offers a more lateral trajectory with improved access to the petrous apex with decreased need for manipulation of the ICA (29). Therefore, it is possible to increase the GTR safely.

The lower clivus extends from the floor of the sphenoid body to the foramen magnum and is shorter but difficult to access (6, 9). The lower part of the petroclival fissure, the jugular foramen, and the foramen magnum were at the level of the lower clivus. The SBCs located in these spaces have the lowest GTR rate, whether with open surgery or EES (2, 19, 23). We can resect the SBCs located above the body of C2 by EES (30). While for the tumors extending to lower than C2, which is regarded as below the inferior limitation of EES, we will use EES combined with transoral approach or purely use the transoral method. At our initial EES stage, we performed the midline linear incision on the nasopharyngeal and pulled the mucous membranes and muscles laterally. However, the exposed field is small. It resulted in the meager GTR rate previously. After that stage, we excised the nasopharyngeal muscle-mucosal tissue, which improved the resection. However, the local infection and CSF leakage limited the GTR of SBCs. Recently, we use the inverted U-shaped rhinopharyngeal flap (31). We once hypothesized that the rhinopharyngeal flap would help improve the skull base reconstruction, just like the initial hypothesis of Champagne et al.; however, their recent retrospective analysis suggests that the rhinopharyngeal flap may not help to reduce CSF leakage (31). This result needs further prospective studies with a larger sample size, and we still use the rhinopharyngeal flap combined with pedicled nasal septal flap for skull base reconstruction. The larger inferior turbinate, eustachian tube, and pterygoid plates limit access to the tumors and the vital structure located lateral region (28). Drilling these bone structures and resecting the eustachian tube supply a more sideways view. However, the resection of the eustachian tube may cause hearing damage and otitis media with effusion, which may need to be dealt with by surgery. Recently, Labib et al. introduced that the surgical skill with the anterolateral mobilization of the eustachian tube provides excellent access to the ventral jugular foramen and infra-petrous region (32). This technique may minimize the complication caused by eustachian tube injury.

CSF leakage was one of the most common complications in EES surgery. In the present series, the tumors in the nine patients with CSF leakage invaded the subdural space, and the tumor volume was large, and five of them were recurrent tumors. Furthermore, two patients have RT history and underwent delayed CSF leakage. Hence, the reconstruction of the skull base is crucial for improving the quality of SBCs surgery, especially in patients with recurrent SBCs and RT history. ICA injury is the most urgent and dangerous complication in EES treatment of SBCs, related to the close relationship between tumors and ICA. Two patients in this series suffered from ICA injuries. These two patients both experienced surgical and RT history previously, and the tumor texture was hard. DSA and balloon occlusion test in patients with a high risk of ICA injury are helpful for preoperative evaluation, and endovascular treatment provides a pivotal safeguard for ICA injuries during EES (2, 33). Simply put, the complications are low in the current series, which may represent the safety of the surgical strategy and be associated with the fact that our group had experienced a long learning curve in EES. Furthermore, navigation, intraoperative Doppler, and intraoperative electrophysiology monitor have considerably improved surgical safety (12, 21).

The limitation of our study is that the follow-up is short and that we cannot deduce the correlation between the surgical resection and prognosis. However, previous studies have suggested that the quality of surgery is crucial for post-surgical outcomes (15, 23). To our knowledge, this study included the largest number of patients in a short period. However, the number of primary SBC patients was still not big enough to get a statistical difference between the derivation group and the validation group. We continue to use the strategy to resect SBC now, and it may lead to the finding of statistical difference with the increasing number of the primary SBCs. Another limitation is that we did not analyze the pathological and genomic features, and these characteristics are correlated with the recurrence (3, 16, 34). These characteristics may indicate the origin and growth direction of SBCs and may be associated with the degree of resection.

## Conclusion

Our results support the concept that SBCs extend along the bone suture and often invade the eight spaces mentioned above (i.e., DS-PCP, bilateral posterior spaces of cICA, bilateral posterior spaces of pc-la ICA, bilateral petroclival fissure spaces that extend toward the medial part of the jugular foramen, and FM-CCJ). Based on this extending character, the surgical strategy introduced in the present study is to explore the hidden spaces sequentially using the EES techniques. This strategy potentially improves surgical resection and decreases residue. This strategy is applicable in both primary SBCs and recurrent SBCs.

## Data Availability Statement

The original contributions presented in the study are included in the article/supplementary material. Further inquiries can be directed to the corresponding authors.

## Ethics Statement

The studies involving human participants were reviewed and approved by the Ethics Review board of Beijing Tiantan Hospital. Written informed consent to participate in this study was provided by the participants’ legal guardian/next of kin.

## Author Contributions

JB, ML, CZL, and YZ designed the study. JB and ML wrote the manuscript and performed the analysis. YX, YS, CHL, PZ, LC, and SG contributed to data collection, manuscript discussion, figures, and tables. All authors contributed to the article and approved the submitted version.

## Funding

This study was supported by the National Natural Science Foundation of China (81771489, 82071559, and 82072804).

## Conflict of Interest

The authors declare that the research was conducted in the absence of any commercial or financial relationships that could be construed as a potential conflict of interest.

## Publisher’s Note

All claims expressed in this article are solely those of the authors and do not necessarily represent those of their affiliated organizations, or those of the publisher, the editors and the reviewers. Any product that may be evaluated in this article, or claim that may be made by its manufacturer, is not guaranteed or endorsed by the publisher.
